# How Fear of COVID-19 Affects Service Experience and Recommendation Intention in Theme Parks: An Approach of Integrating Protection Motivation Theory and Experience Economy Theory

**DOI:** 10.3389/fpsyg.2022.809520

**Published:** 2022-02-28

**Authors:** Yu Pan, Jing (Bill) Xu, Jian Ming Luo, Rob Law

**Affiliations:** ^1^Faculty of International Tourism and Management, City University of Macau, Macau, Macau SAR, China; ^2^College of Professional and Continuing Education, Hong Kong Polytechnic University, Hong Kong, Hong Kong SAR, China; ^3^Asia-Pacific Academy of Economics and Management, Department of Integrated Resort and Tourism Management, Faculty of Business Administration, University of Macau, Macau, Macau SAR, China

**Keywords:** fear of COVID-19, perceived risk, participation, service experience, recommendation intention, protection motivation theory, experience economy theory

## Abstract

The unprecedented public panic caused by COVID-19 will affect the recovery of tourism, especially the theme parks, which are generally crowded due to high visitor volume. The purpose of this study is to discuss the effect of the COVID-19 on the theme park industry. This study aims to predict recommendation intentions of theme park visitors by exploring the complicated mechanism derived from the fear of COVID-19. This study uses a quantitative research method, and SPSS 20.0 and AMOS 22.0 were used for data analysis. An online survey was conducted with 420 Chinese respondents who visited Shanghai Disneyland after its reopening. The study explored the relationship between Fear of COVID-19, perceived risk, participation, service experience, and revisit intention. Results indicated the perceived risk of theme park visitors will not directly ruin their recommendation intention. Visitors’ fear of COVID-19 enhanced their perceived risk, reduced their desire for active participation and impaired their service experience, which consequently affected their recommendation intention. We provide theoretical and managerial implications.

## Introduction

The global tourism industry has suffered a contraction of over 80% of businesses since the COVID-19 pandemic surfaced (UNWTO, 2020). Epidemiologists and medical experts believe that the COVID-19 could become a persistent global pandemic. The long-term effects of COVID-19 on the tourism industry are considerably more serious than the effects of SARS in 2003 and MERS in 2012 (Baldwin and di Mauro, 2020). Pandemic regulations and control measures, such as maintaining social distance and restricting people’s gatherings, have severely affected tourism sectors, such as airlines, hotels, theme parks, and attractions. Among them, theme parks and attractions, before COVID-19, have substantially contributed to tourism economies (Dong and Siu, 2013). They provide core entertainment activities, such as themed facilities, rides, and various supplementary services (e.g., food and beverages, shopping, and other services), thereby creating novel and pleasurable experiences for tourists and visitors.

The theme park industry has been expanding globally, attracting thousands of tourists annually. In 2019, the top 10 theme parks had 521.18 million tourists, an increase of 4% compared with the same period in 2018 (TEA, 2019). Unfortunately, theme parks were also affected by COVID-19 because they represent densely populated places and must deal with operational and marketing risks associated with the pandemic. Data from the China Theme Park Research Institute indicated that 2,075 theme parks in China have been closed since the emergence of COVID-19 (Yang, 2020). However, stringent pandemic prevention and control measures have eventually led to the reopening of numerous theme parks since April 2020 (Jolly, 2020). Despite their reopening, the effects of COVID-19 persist, and many theme park visitors may still fear COVID-19, which will affect their experiences and following behaviours (Wen et al., 2020).

One popular theory of cognitive psychology related to risk perception and people’s negative emotions (Rather, 2021a) is the protection motivation theory (PMT) (Rogers, 1975). The theory indicates that people manifest protection and risk prevention behaviours after their assessment of external threats (or negative events) and the anticipation of negative consequences. Here, a natural outcome of threat appraisal in PMT is people’s negative emotions (Zheng et al., 2021). Previous studies have explored considerably the role of customers’ emotions, particularly positive emotions for marketing and management purposes (Addis et al., 2018). However, academic attention to negative emotion is insufficient, for example, in tourism (Nawijn and Fricke, 2015). Currently, fear of COVID-19 is an outstanding negative emotion. Recent studies have attempted to explore tourists’ fear of COVID-19 (Das and Tiwari, 2020; Jian et al., 2020; Luo and Lam, 2020) and the corresponding risk perceptions (Abraham et al., 2021; Hao et al., 2021) on their behavioural intention. However, these studies did not fully attach importance to the complicated mechanism in the service consumption setting that the fear and perceived risk in a pandemic environment will first affect the experiences of tourists who may then decide to behave accordingly, such as whether to recommend the tourism services or not.

In some tourism services (e.g., theme parks), visitors often expect a high degree of participation (Wu, 2011). Based on experience economy theory (EET) (Pine and Gilmore, 1999), active participation is a prerequisite of optimal experiences, and in our study, service experience in tourism (Xu and Chan, 2010). However, in a pandemic environment, visitors’ participation may be remarkably affected when they perceive risks and tend to protect themselves. For example, they may desire not to visit crowded places inside a theme park and avoid group activities and close interactions with service staff or other visitors (Wen et al., 2020). Thus, the relationship between visitors’ fear of COVID-19, perceived risk and their service experiences can be complicated, which has not been proven in the extant literature. Thus, we aim to address this knowledge gap by taking an integrated approach to applying PMT and EET. Moreover, although EET has been widely applied in tourism (Ketter, 2018), PMT appears to be inadequately given credit (Wang et al., 2019) and has not been adopted together with EET to shed light on the understanding of tourists’ participation and service experiences.

Lastly, visitors without pleasurable service experiences may not recommend the service to others (Chen et al., 2020). Currently, word-of-mouth recommendation is becoming considerably valuable because of the expected decreasing tourism flows due to the negative public sentiment on COVID-19. Thus, visitors’ recommendation intentions should be predicted by exploring and understanding the effects of fear of COVID-19 in related service consumption settings. Given the paucity of studies in this area, the current study aims to fill the research gap by investigating how fear of COVID-19 further affects service experiences and recommendation intentions. Our three research objectives are as follows:

(1) To explore how fear of COVID-19 results in visitors’ perceived risks in theme parks;
(2) To examine how visitors’ perceived risk further affects their participation and service experiences towards their recommendation intention; and
(3) To provide managerial insights and advice to managers of theme parks or other tourist attractions on the importance of managing the negative emotions of visitors.

## Literature Reviewed

### Protection Motivation Theory and Experience Economy Theory

Protection motivation theory was proposed by Rogers (1975) to explain individuals’ cognitive processes when facing a threat. PMT assumes that when exposed to a threatening event, people may decide to participate in risk prevention behaviours to protect themselves. They may go through processes of “threat appraisal” and “coping appraisal” (Rogers, 1975; Shafiei and Maleksaeidi, 2020). The former involves an evaluation of the degree of the threat (i.e., severity) and the probability that it may occur (i.e., vulnerability), whereas the latter revolves around coping behaviours. Despite the recorded applications of PMT in tourism, one could argue that it is insufficient (Wang et al., 2019). For example, based on Wang et al.’s (2019) summary of tourism studies using PMT, particularly those in the recent decade (Schroeder et al., 2013; Fisher et al., 2018; Zheng et al., 2021), most studies have tended to focus on predicting tourists’ behavioural intentions without looking through tourists’ threat appraisals and coping behaviours in a negative relationship with their experiences. Our study attempts to fill this gap by integrating PMT and EET (Pine and Gilmore, 1999).

Experience economy theory was developed by Pine and Gilmore (1999) based on the understanding that offering an optimal and memorable experience for consumers is necessary in the contemporary economy. Accordingly, tourists are likely to seek an entertaining, educational, aesthetic, and escapist experience, and their active participation in the tourism activities (Mereu, 2016). However, despite the plethora of applications of this theory in tourism (Ketter, 2018; Lee et al., 2020), studies assessing tourists’ experiences while perceiving certain threats and risks in the service environment remain scarce.

Previous studies have mainly focused on the impact of negative emotion caused by COVID-19 on motivation, attitudes, and behavioural intentions in the tourism industry (Matiza, 2020; Sigala, 2020; Rather, 2021a; Sánchez-Cañizares et al., 2021). In the context of the epidemic, while the concept of innovative technology for service experience is widely discussed in the hospitality industry (Rahimizhian and Irani, 2020; Kim et al., 2021), it is rarely discussed for the theme parks industry with a focus on participation and experience. This study fills the research gap by investigating how fear of COVID-19 further affects service experiences.

In the present study, our theoretical contributions centre on the application of both theories by examining the inter-relationships among PMT-related constructs (fear of COVID-19, perceived risk), EET-related constructs (participation, service experience) and behavioural intention (i.e., recommendation intention as the proxy) in a new conceptual framework. Fear of COVID-19 was chosen as an outcome of people’s appraisal of COVID-19 threat in general (Rather, 2021a) while perceived risk was also highlighted to assess the level of risk for a theme park visit. Previous PMT-based studies have used it as a core in their conceptual frameworks (Qi et al., 2009; Schroeder et al., 2013). Participation and service experience were chosen to shed light on the mechanism of forming theme park experiences alongside applying EET, because the former represents a prerequisite of the latter (Pine and Gilmore, 1999; Dong and Siu, 2013). Connecting PMT to EET further, it is believed that in theme parks, if visitors have the fear of COVID-19 and perceive high risk in visiting a theme park, their participation and service experiences will be affected because of the chosen protective and coping behaviours (Rather, 2021a). The following sections review relevant literature concerning fear of COVID-19 and perceived risk, and then those about participation, service experience, recommendation intention, and interrelationships among different constructs.

### Fear of COVID-19

Fear is a negative emotion that represents an individual mental response to threatening stimuli in the environment (de Hoog et al., 2008). Cisler et al. (2009) explained that fear is the main emotion of people when faced with threats of uncertainty. In tourism, Kang et al. (2012) noted that fear can significantly affect tourists’ activities and destination choices. Luo and Lam (2020) further argued that tourists’ fear negatively affects travel intention.

Epidemics refer to highly infectious and rapidly spreading diseases (Strong, 1990). Because of the uncertainty and possibility of fatal consequences of the new virus, the fear of the virus spread rapidly in public (Person et al., 2004). COVID-19 has been an ongoing pandemic with over 200 million COVID-19 cases reported worldwide at the time of this study (JHU CSSE, 2021). The psychological impact of COVID-19 on everyone has been profound; that is, it inevitably triggers significant fear of health and death amongst people (Elemo et al., 2020). First, this type of fear could spread rapidly in public possibly because of the lack of knowledge and the uncertainty and possibility of its fatal consequences (Person et al., 2004). Second, during such pandemics, governments often implement coercive measures, such as travel restrictions, banning public activities, closing schools, quarantine, and isolation measures, which may further exacerbate the fear (Eichelberger, 2007). Because of the current popularity of social media, one’s fear of the pandemic may also be aggravated by observing others’ online reviews (Dalrymple et al., 2016). Fear of infection and uncertainty causes people to suffer from a higher degree of mental health problems and also affects people’s participation in social activities (Quadros et al., 2021). Currently, the assumption is that fear of COVID-19 may be long-lasting, and may be something that people, and tourists, in particular, must deal with constantly (Hall et al., 2020).

In line with PMT, fear of COVID-19 can be regarded as an emotional outcome of people’s “threat appraisal” of COVID-19 in general (Rather, 2021a; Zheng et al., 2021). According to Rad et al. (2021), an individual’s fear level will increase if someone feels vulnerable to COVID-19’s serious health threat, which further motivates him or her to adopt preventive or protective behaviours. However, the role and effect of fear of COVID-19 still requires further research and evidence in different contexts (Rather, 2021b). In our study, we argued that one visitor’s fear of COVID-19 will determine his or her protective behaviours and experiences in theme parks through an evaluation of perceived risks.

### Perceived Risk

In cognitive psychology, perceived risk refers to the perception of uncertainty and potential threats (Hao et al., 2021). Risk perception can be interpreted as a subjective assessment of the risks subject to their characteristics and severity of the threats (Moreira, 2008). It has been widely studied in the research on tourist behaviour (Kim et al., 2016). When travelling, tourists face various risks pertaining to their health conditions, external natural disasters, and political and crime problems at destinations (Giusti and Raya, 2019). Tourists’ perceived risk is arguably dependent upon their risk assessment and affects their decision-making related to consuming tourism products (Reisinger and Mavondo, 2015). In line with PMT, perceived risk shares a conceptual foundation with “susceptibility” or “vulnerability” (Qi et al., 2009; Schroeder et al., 2013). In our study, perceived risk was defined to reflect visitors’ overall risk evaluation for a theme park visit based on their understanding that theme parks are vulnerable to COVID-19.

Tourists’ risk perception can be influenced by multiple factors, including external factors, such as the media, other information sources and surrounding influence groups, as well as internal factors inclusive of personal cultural backgrounds, past experiences (Lepp and Gibson, 2003) and affective states, such as fear (Godovykh et al., 2021). The COVID-19 pandemic is a public health threat that exacerbates mental health stress through fear, panic, and uncertainty, thereby increasing people’s psychological risk and vulnerability (Mamun, 2021a,b). The spread of the COVID-19 has brought severe environmental uncertainty, which has led individuals to become markedly cautious with risk-taking (Amin, 2020). Alternatively, fear of COVID-19 is arguably believed to enhance people’s perceived risk for service consumption (Szymkowiak et al., 2021). This linkage will be strengthened because of the nature of theme parks which are built to attract a high volume of visitors in daily operations while requesting a great level of manpower, full-time and part-time, to serve the visitors in the meantime (Milman et al., 2020). Thus, because of the crowdedness and expected close interactions between people (Solmaz et al., 2015), theme parks appear to be highly vulnerable and susceptible to COVID-19, particularly for potential visitors who are afraid of COVID-19 (i.e., fear of COVID-19). Thus, we propose the following hypothesis:

H1: *Fear of COVID-19 affects perceived risk.*

### Service Experience

According to EET (Pine and Gilmore, 1999), customer experience is highlighted by the service providers in the service-dominant industry (Shaw et al., 2011). In the tourism context, tourists and visitors have a desire for memorable and pleasurable service experiences in numerous sectors (Ketter, 2018). Theme parks are a representative sector because of their nature of staged authenticity and hyper-reality. Moreover, theme parks have long been at the forefront of innovative design, marketing and providing memorable experiences to cater to the contemporary trends of leisure and recreation sectors (Geissler and Rucks, 2011). In theme parks, visitors’ service experiences reflect their cognition and emotional evaluation of product and service consumptions (Dong and Siu, 2013; Lee et al., 2020). Specifically, while travelling with their friends, family members or relatives, visitors may want to experience various kinds of rides (e.g., white knuckle, slow, family, water rides), activities and games (e.g., fishing, bungee), one-time “must-see” shows and thematic foods and beverages (Luo et al., 2020). All these components contribute to the overall service experience evaluations in theme parks.

Although service experience is a well-established concept, few studies have explored its barriers. People’s risk perception, as derived from negative emotions, may negatively affect their service experiences (Mbama and Ezepue, 2018). Tourists have proven to be considerably sensitive to their potential encountered risks, and their experiences can be influenced directly by risk perceptions (Lepp and Gibson, 2003). Our study assumed that theme park visitors’ perceived risks directly and negatively influence their service experiences. Accordingly, the higher the perceived risk, the worse the service experiences. Alternatively, the lower the perceived risk, the greater the service experiences. Therefore, we formulate the following hypothesis:

H2: *Perceived risk negatively affects service experience.*

### Participation

Customer participation during service consumption is also evident in line with EET (Chang, 2018). In tourism, tourists expect a high degree of participation when consuming numerous tourism products (Fan et al., 2020). It is a crucial predisposition for customers’ participation and interactions with service providers (Dong and Siu, 2013). It is especially evident given today’s experience economy (Pine and Gilmore, 1999) that tourists expect from their consumption of tourism products.

Theme parks represent a typical tourism sector that visitors are highly inclined to participate intensively while receiving in-park services and experiencing park facilities (Fu et al., 2017). Theme parks have developed and evolved over the years, from an emphasis on passive experience to more interactive experiences, for which visitors are involved as active participants in creating and inputting their own experiences (Baker, 2016). For example, today’s visitors can learn about new cultures and improve their skills and knowledge (e.g., cooking skills) in theme parks (Kauppinen-Räisänen et al., 2013). Many theme park services are also designed to involve visitors in the creation of interactive experiences (e.g., Turtle talk in Disney World) (Oh and Ma, 2018). The socialisation factor has also been found to stimulate theme park visitors’ participation in service (Wu, 2011). This finding is because emotional labours are highly requested in theme parks to stimulate visitors’ participation (Bryman, 1999). Thus, visitors become highly immersed in such interaction and participation-oriented environments in theme parks (Lee et al., 2020).

However, people’s participation in service may be hindered by numerous factors, one of which relates to their perceived risk. Prebensen et al. (2013) used the probability of perceived risk to examine individuals’ involvement. Similarly, people’s participation may significantly decline when they are sensitive to and perceive certain risks in activities (Miles et al., 2008). This phenomenon can be well explained by PMT (Rogers, 1975) in that people who perceive being exposed to an external threat with high risks involved may turn to a low level of participation in services as a reflection of their protective and coping behaviours (Lee, 2011). Our study proposed that if visitors perceive a high risk for the theme park visit and services, their participation will be affected. Specifically, they may not like to visit crowded themed lands and avoid close interpersonal interaction and group activities to a great extent, thereby resulting in a lower level of participation. Thus, we proposed the following hypothesis:

H3: *Perceived risk negatively affects participation.*

Inevitably, participation in services is related closely to service experiences (Xu and Chan, 2010). Elsharnouby and Mahrous (2015) indicated that pleasurable service experiences depend on customers’ active participation. Customers can co-create consumption values and service experiences with a high degree of participation (Dong and Siu, 2013). In our study, theme park visitors normally expect to be involved deeply in this kind of hyper-real service environment and have intense interactions with service providers and performers (Lee et al., 2020). Their participation relates positively to their service experiences. Thus, we propose the following hypothesis:

H4: *Participation positively influences service experience.*

### Recommendation Intention

Recommendation, also known as “word-of-mouth recommendation,” is the dissemination of positive comments on specific objects, services or organisations (Chien et al., 2018). Because word-of-mouth is usually regarded as a reliable information source, the effects of customers’ recommendations is usually stronger than traditional advertising (Nguyen and Romaniuk, 2014). Fullerton (2005) believed that consumers’ recommendations can create profits for enterprises. The recommendations could help enterprises attract new customers. Because of COVID-19, tourists’ loyal behaviours may be difficult to perceive through revisit behaviours (Hassan and Soliman, 2021). Instead, their recommendations to other potential visitors may matter substantially in the future, which service providers must emphasise.

In this study, recommendation intention was chosen as the proxy of behavioural intention. It refers to the intention of dissemination of positive comments on specific objects, services or organisations (Chien et al., 2018). In our study, despite the lack of future revisit intention because of COVID-19, theme park visitors could consider spreading recommendation messages through oral, online or other communication channels after retaining memorable experiences in a theme park visit (Ma et al., 2017). We also assumed that a theme park visitor’s recommendation intention would be influenced by three factors: perceived risk, participation, and service experience.

First, perceived risk can directly change one’s behavioural intention, including recommendation intention (Al-Ansi et al., 2019). Cong (2021) conducted an empirical study of European tourists in Vietnam and found that the greater the perceived risk by the tourists, the more negative they are about their recommendation intention. Therefore, we assumed similarly in theme parks that when visitors perceive high risk, they will have already felt reluctant to recommend. Thus, we formulated the following hypothesis:

H5: *Perceived risk negatively influences recommendation intention.*

Second, other than the negative influence of perceived risk, we also considered two positive predictors of recommendation intention. Among the two, participation should be a prerequisite for recommendation intention (Chang et al., 2018) particularly in those participation-oriented service sectors, e.g., theme parks (Lee et al., 2020). Some studies have indicated that active consumers may invite others to participate in certain service consumption activities (Heinonen, 2011; Chen et al., 2017). Furthermore, according to Chen et al. (2017), in social media, active consumers may share knowledge and experiences with other consumers and even invite them to participate in the consumption. In our study, we also assumed that theme park visitors’ active participation relates positively to their recommendation intention. Hence, we propose the following hypothesis:

H6: *Participation positively influences recommendation intention.*

Lastly, the other positive predictor of recommendation intention is service experience, which has been widely proven in literature. For example, Altunel and Erkurt (2015) found that tourist experience positively influences recommendation intention in culture tourism. Another study indicated that the festival experience at a destination will lead to the recommendation intention for the destination (Culha, 2020). In theme parks, it has also been known that if visitors are satisfied with their service experiences, they will recommend them to other potential visitors (Ma et al., 2017). Thus, in our study, it is assumed that theme park visitors’ recommendation intentions are derived from their service experiences. Thus, we formulated the following hypothesis:

H7: *Service experience positively influences recommendation intention.*

### Conceptual Model

**FIGURE 1:**
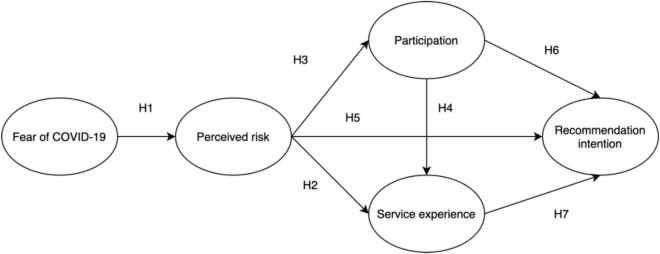
Conceptual model.

This study used the preceding literature review and hypothesis development as bases to propose that theme park visitors’ fear of COVID-19 will affect their service experiences through perceived risks and will affect their participation and recommendation intention. With the integration of PMT and EET, we proposed our conceptual model (Figure 1).

## Materials and Methods

### Measures

The measurement scales of the existing research were used in this study, which have been proven effective and valid, to develop the questionnaire. Fear of COVID-19 was measured based on Ahorsu et al. (2020) and Bhuiyan et al. (2020). This study defined perceived risk as the perception of uncertainty and potential threats, it was measured by adopting the scales used by Fuchs and Reichel (2006). Based on the reality that theme park visitors are the subject of this study, the measurement of theme park visitor service experience in this study using the theme park visitor service experience scale of Dong and Siu (2013). Due to the same research background, scales from Dong and Siu (2013) were adapted to evaluate theme park visitors’ participation. Following Altunel and Erkurt (2015), the measure of recommendation intention was developed. These items are appropriate in the setting of tourism. Since prior research have applied similar items in determining tourists’ recommendation intention in tourism. The preceding measures were anchored a 7-point Likert scale, ranging from “strongly disagree” (1) to “strongly agree” (7). To ensure accuracy, these items were translated from English to Chinese, and then revised and translated back into English. The measurement scales were checked by bilingual scholars to ensure the equivalence of the questionnaire’s Chinese and English versions. Marketing and tourism scholars helped examine the validity of the questionnaire. In the final version, 22 scale items were used to measure the focal variables. Other questions requested the demographic information of the participants.

### Data Collection

The target survey respondents of this study were theme park visitors. Because of the current pandemic environment and social distancing requirement, an onsite questionnaire survey is not permitted. Instead, a professional data service company was recruited to conduct an online survey (Fong et al., 2020). In our study, the recruited company was experienced company with an extensive database of Chinese residents (WenJuanXing, 2021). The professional data service company is widely used in scientific research data collection in China (Peng et al., 2019; Shi et al., 2021). A simple random sampling method was employed (Acharya et al., 2013) and the company sent out the questionnaires through its panel database. Tourists, or Chinese visitors regardless of their places of origin, who had visited Shanghai Disneyland after it reopened in May 2020 were treated as qualified respondents.

A pilot study with 50 respondents was conducted, and later, slight modifications were made for the survey items and the questionnaire. In order to resolve the problem of Common method bias, mixing the order of the questions to using different scale types in the online questionnaire (Chang et al., 2010). Then, data for the main survey were collected from November 10, 2020 to December 30, 2020. To ensure the quality of the data, repeated questions were embedded in the questionnaire to check the consistency of the responses (Ye et al., 2021). Finally, 604 questionnaires were distributed, and 420 valid responses were obtained, yielding a valid response rate of 69.54%. This sample size is considered highly satisfactory for our multivariate data analysis purposes (Lai and Hitchcock, 2015).

### Data Analysis

SPSS 20.0 and AMOS 22.0 were used for data analysis. In particular, SPSS 20.0 was used to conduct descriptive analyses. Then, confirmatory factor analysis (CFA) was conducted using AMOS 22.0 to test the measurement model. Reliability and validity were checked following the suggested criteria of Fornell and Larcker (1981). Finally, the structural model was tested to analyse the hypothesised relationships among the constructs (Byrne, 2001).

## Results

### Sample Profile

The sample had 51% female and 49% male visitors. The respondents were between the ages of 18 and 35 years (65%). This result is consistent with the recent visitor profile of Shanghai Disneyland, which attracted more young visitors than seniors because of COVID-19 concerns, as proven by the managerial park staff. Moreover, 55% of the respondents were single and 98% held a university diploma or above. Therefore, our sample is generally representative of the theme park visitors in a pandemic environment (Table 1).

**TABLE 1:** Demographic profile of respondents (*N* = 420).

Variable		Frequency	Percentage
Gender	Male	206	49%
	Female	214	51%
Marital Status	Married	189	45%
	Single	231	55%
Age	18–25 years old	139	33%
	26–35 years old	134	32%
	36–45 years old	101	24%
	46–55 years old	46	11%
	56–65 years old	0	0
	Over 65 years old	0	0
Education	High school or below	8	2%
	Diploma	227	54%
	Degree	164	39%
	Master or above	21	5%
Occupation	Student	206	49%
	Working	189	45%
	Housewife	17	4%
	Retired	8	2%
Personal Monthly Income (RMB)	Less than 5000	84	20%
	5000–9999	138	33%
	10,000–14,999	105	25%
	15,000–19,999	63	15%
	20,000–29,999	17	4%
	30,000 and above	13	3%

### Measurement Model

The CFA results (Table 2) showed that the measurement model is valid because the χ2/df value is 2.423, and comparative fit index (CFI) is 0.965, Tucker–Lewis index (TLI) is 0.919, the goodness of fit index (GFI) is 0.893, and root-mean-square error of approximation (RMSEA) is 0.058 (Byrne, 2001). These results showed that the model is reasonably fit (Byrne, 2001). The model was further verified in terms of reliability, convergent validity, and discriminant validity. Taber (2018) explained that the value of Cronbach’s alphas should be above 0.7, which indicates that the instrument has high reliability. All Cronbach’s alphas in the current study are between 0.907 and 0.958. The composite reliability (CR) of every construct is between 0.958 and 0.913. The highest average variance extracted (AVE) is 0.884, and the lowest value is 0.662, which exceeded the cut-off of 0.5 for convergent validity (Fornell and Larcker, 1981). Furthermore, the square root of each AVE is higher than the correlation of the related pairwise constructs, which also satisfied the requirement of discriminant validity (Fornell and Larcker, 1981; Table 3).

**TABLE 2:** Confirmatory factor analysis results.

Latent variable	Measurement item	Standardised loading	Cronbach’s alpha	CR	AVE	References
Fear of COVID-19 (FC)	Afraid of COVID-19.	0.647	0.931	0.932	0.662	Ahorsu et al., 2020; Bhuiyan et al., 2020
	Uncomfortable to think.	0.776				
	My hands perspire when I think of COVID-19.	0.862				
	Afraid of losing my life.	0.832				
	When watching news and stories about COVID-19 on social media or any other media (i.e., TV, Radio), I become nervous or anxious.	0.836				
	Cannot sleep because I am worried about being infected with COVID-19.	0.813				
	My heart races or palpitates when I think about being infected with COVID-19.	0.904				
Perceived risk (PR)	I thought that my friends or relatives would worry about my safety whilst I was in the theme park.	0.644	0.927	0.928	0.724	Fuchs and Reichel, 2006
	Prior to my trip, I view the theme park as more dangerous than other tourist attractions.	0.873				
	Theme parks are risky.	0.934				
	Friends or relatives see the theme park as a risky place to visit.	0.890				
	Theme park is a dangerous place for tourists.	0.884				
Participation (PA)	Totally involved in the theme park.	0.920	0.907	0.911	0.775	Dong and Siu, 2013
	Quite absorbed in the experience.	0.933				
	Nearly forgot the time when I was playing.	0.780				
Service experience (SE)	Satisfied with the whole experience.	0.948	0.958	0.958	0.884	Dong and Siu, 2013
	Memorable.	0.911				
	Enjoyable.	0.961				
Recommendation intention (RI)	Say positive things about this theme park to other people.	0.877	0.911	0.913	0.724	Altunel and Erkurt, 2015
	Recommend it to someone who seeks my advice.	0.900				
	Encourage friends and relatives to visit the theme park.	0.824				
	Share the experience with my friends and relatives.	0.800				

*AVE, average variance extracted.*

**TABLE 3:** Fornell–Larcker criterion.

	Fear of COVID-19	Perceived risk	Participation	Service experience	Recommendation intention
Fear of COVID-19 (FC)	0.814				
Perceived risk (PR)	0.532	0.850			
Participation (PA)	–0.148	–0.123	0.880		
Service experience (SE)	–0.225	–0.237	0.520	0.940	
Recommendation intention (RI)	–0.203	–0.207	0.554	0.540	0.850

*The gray meaning is the AVE under the root. The following is the correlation.*

### Structural Model

**FIGURE 2:**
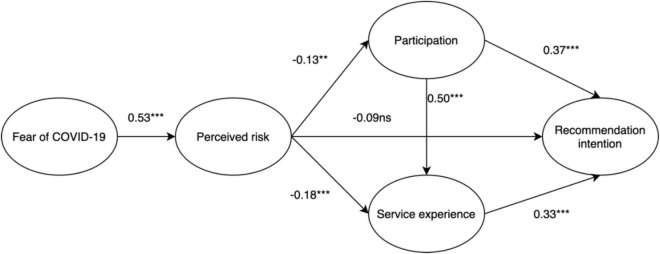
Structural model. **p < 0.01; ***p < 0.001; ns, non-significant.

The maximum likelihood method was adopted to examine the structural model with inter-relationships amongst the five constructs (i.e., fear of COVID-19, perceived risk, service experience, participation, and recommendation intention). The results of model fit indices are acceptable (Figure 2). The χ2/df value is 2.421, and CFI is 0.964, Tucker–Lewis index (TLI) is 0.959, the goodness of fit index (GFI) is 0.893 and root-mean-square error of approximation (RMSEA) is 0.058 (Byrne, 2001). Table 4 shows the results of the hypothesis testing. The results indicated that fear of COVID-19 affects perceived risk (β = 0.533, *p* < 0.01); perceived risk negatively affects service experience (β = –0.178, *p* < 0.001); perceived risk negatively affects participation (β = –0.126, *p* < 0.01); participation positively influences service experience (β = 0.497, *p* < 0.01); perceived risk negatively affects recommendation intention (β = –0.086, *p* > 0.05); participation negatively affects recommendation intention (β = 0.374, *p* < 0.001) and service experience positively influences recommendation intention (β = 0.325, *p* < 0.001). Thus, six hypotheses are supported, one hypothesis (H5) is rejected.

**TABLE 4:** Structural model test.

Path	Path coefficients	*t*	*p*
Fear of COVID-19 (FC) ⇒ Perceived risk (PR)	0.533	9.570	<0.001
Perceived risk (PR) ⇒ Service experience (SE)	–0.178	–3.943	<0.001
Perceived risk (PR) ⇒ Participation (PA)	–0.126	–2.418	0.016
Participation (PA) ⇒ Service experience (SE)	0.497	10.868	<0.001
Service experience (SE) ⇒ Recommendation intention (RI)	0.325	6.282	<0.001
Perceived risk (PR) ⇒ Recommendation intention (RI)	–0.086	–1.954	0.051
Participation (PA) ⇒ Recommendation intention (RI)	0.374	7.207	<0.001

Our results also showed that the indirect effect of fear of COVID-19 on recommendation intention was significant (β = –0.113) (Table 5). Fear of COVID-19 also has an indirect effect on service experience (β = –0.129) and on participation (β = –0.067). After 5000 bootstrapping, the 95% confidence interval (CI) is [–0.187, –0.046]. The CI value does not include zero, which indicates that the mediating effect is significant (Cheung and Lau, 2008).

**TABLE 5:** Results of mediation tests.

Causal relationships	Direct effect	Indirect effect	Total effect
FC ⇒ PR	0.533	–	0.533
PR ⇒ PA	–0.126	–	–0.126
PR ⇒ SE	–0.178	–0.063	–0.241
PA ⇒ SE	0.497	–	0.497
SE ⇒ RI	0.325	–	0.325
FC ⇒ RI	–	–0.113	–0.113
FC ⇒ SE	–	–0.129	–0.129
FC ⇒ PA	–	–0.067	–0.067
PR ⇒ RI	–0.086	–0.126	–0.211
PA ⇒ RI	0.374	0.162	0.536

*– means not applicable. FC, fear of COVID-19; PR, perceived risk; PA, participation; SE, service experience; RI, recommendation intention.*

## Discussion and Conclusion

COVID-19 triggers negative emotions that hardly disappear in a short time, which could also hinder the recovery of the tourism industry in the post-pandemic era. Our study focused on clarifying the underlying mechanism of fear of COVID-19 on tourists’ experiences and behaviours. To achieve this objective, we developed a conceptual model and verified it through the survey of theme park visitors. The survey results demonstrated that visitors’ fear of COVID-19 enhanced their perceived risk, reduced their desire for active participation and impaired their service experience, which consequently affected their recommendation intention. Except for H5, the other six hypotheses (i.e., H1, H2, H3, H4, H6, H7) were supported.

Risk and safety were still the central concerns for tourists, which is consistent with previous studies (Abraham et al., 2021). This concern is heightened by the ongoing COVID-19 pandemic and the corresponding public health crisis (Matiza, 2020). We found that fear of COVID-19 positively affects perceived risk (H1), and perceived risk significantly affects service experience and participation negatively (H2 and H3). These results are parallel with previous findings (Miles et al., 2008; Mbama and Ezepue, 2018). Meanwhile, visitors’ participation positively affects the service experience (H4). The current study revealed that customer involvement and participation can enhance service experience, which is highly expected in an entertainment environment and selected tourism sectors (Dong and Siu, 2013). However, our result is inconsistent with previous studies that supported the view that perceived risk negatively influences recommendation intention (thereby rejecting H5) (Cong, 2021). By the same token, the perceived risk of theme park visitors will not directly ruin their recommendation intention but will indirectly affect it through the effects on participation and service experiences. This is especially true for visitors who may have more objective assessment and rational behavioural intention as a result of their own experiences (Ma et al., 2017). Our study unveiled that participation and service experiences could result in visitors’ recommendation intentions directly (H6, H7). This result echoed previous research findings (Altunel and Erkurt, 2015; Jiménez-Castillo and Sánchez-Fernández, 2019; Culha, 2020). The current study indicated that visitors’ fear of COVID-19 enhanced their perceived risk, reduced their desire for active participation and impaired their service experience, which consequently affected their recommendation intention.

### Theoretical Implications

This study contributes to PMT and EET literature in tourism and those of tourist emotion and service experience in the following ways. First, our study applies and integrates PMT and EET. Theme park visitors’ threat appraisals (i.e., fear of COVID-19, perceived risk) of PMT (Rogers, 1975; Rather, 2021a) have been proven to arguably lead to their protection and risk prevention behaviours, which revolve around a choice of more passive participation into services and activities. The lack of active participation affects the formation of optimal experiences following the argument of the EET (Pine and Gilmore, 1999). This innovative approach to theoretical integration also fills a knowledge gap because many recent COVID-19-focused studies (see Liu et al. (2021)) have focused on relevant tourism phenomena from another classical theory: theory of planned behaviour (TPB). One of the main limitations of TPB is that it does not include other powerful measures that may predict behaviour better than the TPB ones in certain circumstances (Sniehotta et al., 2014), such as fear in a pandemic environment.

Second, there was a dearth of tourist studies that focused on uncovering the effects of negative emotion (Nawijn and Fricke, 2015). Prior limited studies have focused on the influence of tourists’ fear as a negative emotion on their travel decisions (Jian et al., 2020; Luo and Lam, 2020). Few studies have evaluated the effects of tourists’ fear on their service experiences and behaviours. COVID-19 triggers fear, specifically in densely populated tourism venues, such as theme parks (Milman et al., 2020). During a pandemic, how tourists’ and visitors’ fear and risk perceptions affect their experiences should be studied. Our study fills the gap in this regard.

Third, theme parks are entertainment venues that stimulate visitors’ high level of participation to generate pleasurable experiences (Wu, 2011; Lee et al., 2020). However, limited studies have attempted to learn the mechanism of how fear and risk perception affects service experiences through the visitors’ participation. We successfully exemplified that theme park visitors’ fear of COVID-19 results in perceived risks, which can further influence service experiences by altering their participation levels. Aligned with PMT, visitors’ self-protection and COVID-19 threat coping behaviours are embedded in their choice of participation level and types (Wang et al., 2019). In other words, theme park visitors might avoid or reduce close interactions with others when they attend different activities and programmes in theme parks.

Lastly, because of the expected reduction in tourism flows and declining revisit intentions caused by the negative public sentiment on COVID-19 (Rather, 2021a), recommendation intention will become an important indicator of tourist loyalty in future tourism. Therefore, we added to the existing knowledge by exploring and understanding the role of perceived risk, participation and service experiences in predicting recommendation intention, either negatively or positively.

### Managerial Implications

Given the fear brought by COVID-19, some effective strategies should be adopted to alleviate this negative emotion and promote tourists’ safety and peace of mind. Theme parks could strengthen pandemic prevention and control measures, such as mask-wearing, flow control, temperature measurement and other means to reduce the fear of COVID-19 and the perceived risks. The frontline service personnel of theme parks consider providing personalised service to make visitors perceive comfort and ensure hygiene and safety control, which may improve visitors’ participation and ensure a pleasurable experience. Moreover, theme parks should communicate with visitors effectively and convey relevant safety information and practices in various forms. For example, pandemic prevention slogans and measures in theme parks can be presented using cartoon images. The intimacy brought by cartoon images to visitors can eliminate the negative cues of risk perception.

Theme parks should also control and promote uncrowded themed lands and service environments. More effective flow management with guest distribution inside parks should be updated during and after COVID-19 pandemic. More virtual queues should be used in place of physical queues to avoid gatherings (Brown et al., 2013).

Visitors’ participation should be activated and assured in a smarter way. For example, individual activities can be promoted more than group activities because the visitors may not have such a high level of self-protection concern. High technology, such as robot services, can be incorporated more to replace interpersonal services (Luo et al., 2021). For example, robots can be presented in the form of cartoon or movie characters and support social distancing practices, thereby minimising the risk of the virus spreading in theme parks. Virtual and augmented reality (VR and AR) innovations can also be incorporated into the design of rides and services to help visitors participate (Wei et al., 2019). For example, Universal Studios Japan has merged AR technology in their new rides that people can participate in competing for the race (Mileva, 2021). Ultimately, in-time feedback systems can be designed and implemented to collect the feedback and advice of in-park visitors for their perception of these innovative designs and service practices.

Lastly, we suggest inviting experienced visitors to help spread the recommendation messages to others and assist in mitigating the fear and perceived risk of potential theme park visitors. Theme parks could provide incentives to those with successfully referred visits. For example, complimentary tickets or group discounts can be given as a reward for such loyal visitors. This kind of referral and redemption programmes can be promoted and conducted online regularly.

### Limitations and Future Research Directions

This study has several limitations that pave the way for future research opportunities. First, given the practical restrictions, this study focused only on Shanghai Disneyland and its visitors. Thus, the results of this study may not be generalisable to other theme parks in other countries. Future studies can test the theoretical relationships by collecting samples from various branded theme parks in different countries. This approach will reduce cultural and geographical differences. Second, our data may not fully reflect the perceptions of visitors. Future studies could consider a cross-sectional approach to collect real-time elicited emotions and experiences, thereby avoiding memory bias. Third, this study did not categorise first-time and repeat visitors. Future research can compare the differences on their theme park experiences and risk perceptions.

## Data Availability Statement

The original contributions presented in the study are included in the article/supplementary material, further inquiries can be directed to the corresponding author.

## Author Contributions

YP and JL jointly contributed to the development of the research framework. YP collected the data and drafted the manuscript. JX directed the manuscript writing and revised the manuscript. RL edited the manuscript. All authors contributed to the article and approved the submitted version.

## Conflict of Interest

The authors declare that the research was conducted in the absence of any commercial or financial relationships that could be construed as a potential conflict of interest.

## Publisher’s Note

All claims expressed in this article are solely those of the authors and do not necessarily represent those of their affiliated organizations, or those of the publisher, the editors and the reviewers. Any product that may be evaluated in this article, or claim that may be made by its manufacturer, is not guaranteed or endorsed by the publisher.

## References

[B1] Abraham V. Bremser K. Carreno M. Crowley-Cyr L. Moreno M. (2021). Exploring the consequences of COVID-19 on tourist behaviors: perceived travel risk, animosity and intentions to travel. *Tour. Rev.* 76 701–717. 10.1108/TR-07-2020-0344

[B2] Acharya A. S. Prakash A. Saxena P. Nigam A. (2013). Sampling: why and how of it. *Indian J. Med. Spec.* 2 330–333. 10.7713/ijms.2013.0032

[B3] Addis M. Miniero G. Soscia I. (2018). Facing contradictory emotions in event marketing: leveraging on surprise. *J. Consum. Mark.* 35 183–193. 10.1108/JCM-06-2016-1862

[B4] Ahorsu D. K. Lin C.-Y. Imani V. Saffari M. Griffiths M. D. Pakpour A. H. (2020). The fear of COVID-19 scale: development and initial validation. *Int. J. Ment. Health Addict.* 35 1–9. 10.1007/s11469-020-00270-8 32226353 PMC7100496

[B5] Al-Ansi A. Olya H. G. Han H. (2019). Effect of general risk on trust, satisfaction, and recommendation intention for halal food. *Int. J. Hosp. Manag.* 83 210–219. 10.1016/j.ijhm.2018.10.017

[B6] Altunel M. C. Erkurt B. (2015). Cultural tourism in Istanbul: the mediation effect of tourist experience and satisfaction on the relationship between involvement and recommendation intention. *J. Destination Mark. Manag.* 4 213–221. 10.1016/j.jdmm.2015.06.003

[B7] Amin S. (2020). The psychology of coronavirus fear: are healthcare professionals suffering. from corona-phobia? *Int. J. Healthc. Manag.* 13 249–256. 10.1080/20479700.2020.1765119

[B8] Baker C. A. (2016). Creative choices and fan practices in the transformation of theme park space. *Transformative Works* 22 1–1. 10.3983/twc.2016.0974 26273990

[B9] Baldwin R. di Mauro B. W. (2020). *Economics in the Time of COVID-19: A New eBook.* London: VOX CEPR Policy Portal, 2–3.

[B10] Bhuiyan A. I. Sakib N. Pakpour A. H. Griffiths M. D. Mamun M. A. A. (2020). COVID-19-related suicides in Bangladesh due to lockdown and economic factors: case study evidence from media reports. *Int. J. Ment. Health* 35 1–6. 10.1007/s11469-020-00307-y 32427168 PMC7228428

[B11] Brown A. Kappes J. Marks J. (2013). Mitigating theme park crowding with incentives and information on mobile devices. *J. Travel Res.* 52 426–436. 10.1177/0047287512475216

[B12] Bryman A. (1999). The Disneyization of society. *Sociol. Rev.* 47 25–47. 10.1111/1467-954X.00161

[B13] Byrne B. M. (2001). Structural equation modeling with AMOS, EQS, and LISREL: comparative approaches to testing for the factorial validity of a measuring instrument. *Int. J. Test.* 1 55–86. 10.1207/S15327574IJT0101_4

[B14] Chang S. (2018). Experience economy in hospitality and tourism: gain and loss values for. service and experience. *Tour. Manag.* 64 55–63. 10.1016/j.tourman.2017.08.004

[B15] Chang S. J. Van Witteloostuijn A. Eden L. (2010). From the editors: common method variance in international business research. *J. Int. Bus. Stud.* 41 178–184. 10.1057/jibs.2009.88

[B16] Chang Y. C. Yeh T. M. Pai F. Y. Huang T. P. (2018). Sport activity for health!! The effects of karate participants’ involvement, perceived value, and leisure benefits on recommendation intention. *Int. J. Environ. Res. Public Health* 15:953. 10.3390/ijerph15050953 29748459 PMC5981992

[B17] Chen H. Phang C. W. Zhang C. (2017). Inviting strangers to participate in collaborative consumption through mobile app. *Int. J. Hum. Comput. Interact.* 33 523–535. 10.1080/10447318.2016.1275434

[B18] Chen X. Cheng Z. F. Kim G. B. (2020). Make it memorable: tourism experience, fun, recommendation and revisit intentions of Chinese outbound tourists. *Sustainability* 12:1904. 10.3390/su12051904

[B19] Cheung G. W. Lau R. S. (2008). Testing mediation and suppression effects of latent variables: bootstrapping with structural equation models. *Org. Res. Methods* 11 296–325. 10.1177/1094428107300343

[B20] Chien S. H. Wu J. J. Huang C. Y. (2018). “We made, we trust”: coproduction and. image congruence in the food-tourism factories. *Asia Pac. Manag. Rev.* 23 310–317. 10.1016/j.apmrv.2018.01.002

[B21] Cisler J. M. Olatunji B. O. Lohr J. M. (2009). Disgust, fear, and the anxiety disorders: a critical review. *Clin. Psychol. Rev.* 29 34–46. 10.1016/j.cpr.2008.09.007 18977061 PMC2895912

[B22] Cong L. C. (2021). Perceived risk and destination knowledge in the satisfaction-loyalty intention relationship: an empirical study of European tourists in Vietnam. *J. Outdoor Recreation Tour. Res. Plann. Manag.* 33:100343. 10.1016/j.jort.2020.100343

[B23] Culha O. (2020). The effect of food festival quality on place attachment and destination. recommendation intention through festival experience and festival satisfaction: the case of the Didim International Olive Festival. *J. Conv. Event Tour.* 21 1–30. 10.1080/15470148.2020.1775743

[B24] Dalrymple K. E. Young R. Tully M. (2016). “Facts, not fear” negotiating uncertainty on social media during the 2014 Ebola crisis. *Sci. Commun.* 38 442–467. 10.1177/1075547016655546

[B25] Das S. S. Tiwari A. K. (2020). Understanding international and domestic travel intention of Indian travellers during COVID-19 using a Bayesian approach. *Tour. Recreation Res.* 45 1–17. 10.1080/02508281.2020.1830341

[B26] de Hoog N. Stroebe W. de Wit J. B. (2008). The processing of fear-arousing communications: how biased processing leads to persuasion. *Soc. Influ.* 3 84–113. 10.1080/15534510802185836

[B27] Dong P. Siu N. Y. M. (2013). Servicescape elements, customer predispositions and service experience: the case of theme park visitors. *Tour. Manag.* 36 541–551. 10.1016/j.tourman.2012.09.004

[B28] Eichelberger L. (2007). SARS and New York’s Chinatown: the politics of risk and blame during an epidemic of fear. *Soc. Sci. Med.* 65 1284–1295. 10.1016/j.socscimed.2007.04.022 17544189 PMC7130736

[B29] Elemo A. S. Satici S. A. Griffiths M. D. (2020). The fear of COVID-19 scale: psychometric properties of the Ethiopian Amharic version. *Int. J. Ment. Health* 18 1–12. 10.1007/s11469-020-00448-0 33293906 PMC7714254

[B30] Elsharnouby T. Mahrous A. A. (2015). Customer participation in online co-creation experience: the role. of e-service quality. *J. Res. Interact. Mark.* 9 313–336. 10.1108/JRIM-06-2014-0038

[B31] Fan D. X. Tsaur S. H. Lin J. H. Chang T. Y. Tsai Y. R. (2020). Tourist intercultural competence: a multidimensional measurement and its impact on tourist active participation and memorable cultural experiences. *J. Travel Res.* 61 414–429. 10.1177/0047287520982372

[B32] Fisher J. J. Almanza B. A. Behnke C. Nelson D. C. Neal J. (2018). Norovirus on cruise ships: motivation for handwashing? *Int. J. Hosp. Manag.* 75 10–17. 10.1016/j.ijhm.2018.02.001

[B33] Fong L. H. N. Law R. Ye B. H. (2020). Outlook of tourism recovery amid an epidemic: importance of outbreak control by the government. *Ann. Tour. Res.* 86:102951. 10.1016/j.annals.2020.102951 32836566 PMC7247783

[B34] Fornell C. Larcker D. F. (1981). Evaluating structural equation models with. unobservable variables and measurement error. *J. Mark. Res.* 18 39–50. 10.1177/002224378101800104

[B35] Fu X. Kang J. Tasci A. (2017). Self-congruity and flow as antecedents of attitude and. loyalty towards a theme park brand. *J. Travel Tour. Mark.* 34 1261–1273. 10.1080/10548408.2017.1343704

[B36] Fuchs G. Reichel A. (2006). Tourist destination risk perception: the case of Israel. *J. Hosp. Leisure Mark.* 14 83–108. 10.1300/J150v14n02_06

[B37] Fullerton G. (2005). The impact of brand commitment on loyalty to retail service brands. *Can. J. Adm. Sci. Revue Can. Sci. Adm.* 22 97–110. 10.1111/j.1936-4490.2005.tb00712.x

[B38] Geissler G. L. Rucks C. T. (2011). The overall theme park experience: a visitor satisfaction tracking study. *J. Vacation Mark.* 17 127–138. 10.1177/1356766710392480

[B39] Giusti G. Raya J. M. (2019). The effect of crime perception and information format on tourists’ willingness/intention to travel. *J. Destination Mark. Manag.* 11 101–107. 10.1016/j.jdmm.2018.10.003

[B40] Godovykh M. Pizam A. Bahja F. (2021). Antecedents and outcomes of health risk perceptions in tourism, following the COVID-19 pandemic. *Tour. Rev.* 76 737–748. 10.1108/TR-06-2020-0257

[B41] Hall C. M. Scott D. Gössling S. (2020). Pandemics, transformations and tourism: be careful what you wish for. *Tour. Geograph.* 22 577–598. 10.1080/14616688.2020.1759131

[B42] Hao Y. Bai H. Sun S. (2021). How does COVID-19 affect tourism in terms of people’s willingness to travel? Empirical evidence from China. *Tour. Rev.* 76 892–909. 10.1108/TR-09-2020-0424

[B43] Hassan S. B. Soliman M. (2021). COVID-19 and repeat visitation: assessing the role of destination social responsibility, destination reputation, holidaymakers’ trust and fear arousal. *J. Destination Mark. Manag.* 19:100495. 10.1016/j.jdmm.2020.100495

[B44] Heinonen K. (2011). Consumer activity in social media: managerial approaches to consumers’ social media behavior. *J. Consum. Behav.* 10 356–364. 10.1002/cb.376

[B45] JHU CSSE (2021). *(Johns Hopkins University Center for Systems Science and Engineering).* Available online at: https://github.com/CSSEGISandData/COVID-19 (accessed August 19, 2021).

[B46] Jian Y. Yu I. Y. Yang M. X. Zeng K. J. (2020). The impacts of fear and uncertainty of COVID-19 on environmental concerns, brand trust, and behavioral intentions toward green hotels. *Sustainability* 12:8688. 10.3390/su12208688

[B47] Jiménez-Castillo D. Sánchez-Fernández R. (2019). The role of digital influencers in brand recommendation: examining their impact on engagement, expected value and purchase intention. *Int. J. Inf. Manag.* 49 366–376. 10.1016/j.ijinfomgt.2019.07.009

[B48] Jolly J. (2020). *Disney Reopens its Shanghai Theme Park, with Many Restrictions.* Available online at: https://www.theguardian.com/film/2020/may/11/coronavirus-disney-reopens-shanghai-theme-park-with-many-restrictions (accessed April 25, 2021).

[B49] Kang E. J. Scott N. Lee T. J. Ballantyne R. (2012). Benefits of visiting a ‘dark tourism’site: the case of the Jeju April 3rd Peace Park, Korea. *Tour. Manag.* 33 257–265. 10.1016/j.tourman.2011.03.004

[B50] Kauppinen-Räisänen H. Gummerus J. Lehtola K. (2013). Remembered eating experiences described by the self, place, food, context and time. *Br. Food J.* 115 666–685. 10.1108/00070701311331571

[B51] Ketter E. (2018). It’s all about you: destination marketing campaigns in the experience. economy era. *Tour. Rev.* 73 331–343. 10.1108/TR-03-2017-0053

[B52] Kim H. Schroeder A. Pennington-Gray L. (2016). Does culture influence risk. perceptions? *Tour. Rev. Int.* 20 11–28. 10.3727/154427216X14581596798942 30089248

[B53] Kim S. S. Kim J. Badu-Baiden F. Giroux M. Choi Y. (2021). Preference for robot service or human service in hotels? Impacts of the COVID-19 pandemic. *Int. J. Hosp. Manag.* 93:102795. 10.1016/j.ijhm.2020.102795 PMC9998175 36919174

[B54] Lai I. K. W. Hitchcock M. (2015). Importance–performance analysis in tourism: a framework for researchers. *Tour. Manag.* 48 242–267. 10.1016/j.tourman.2014.11.008

[B55] Lee S. Jeong E. Qu K. (2020). Exploring theme park visitors’ experience on satisfaction and revisit intention: a Utilization of experience economy model. *J. Qual. Assur. Hosp. Tour.* 21 474–497. 10.1080/1528008X.2019.1691702

[B56] Lee Y. (2011). Understanding anti-plagiarism software adoption: an extended protection motivation theory perspective. *Decis. Support Syst.* 50 361–369. 10.1016/j.dss.2010.07.009

[B57] Lepp A. Gibson H. (2003). Tourist roles, perceived risk and international tourism. *Ann. Tour. Res.* 30 606–624. 10.1016/S0160-7383(03)00024-0

[B58] Liu Y. Shi H. Li Y. Amin A. (2021). Factors influencing Chinese residents’ post-pandemic outbound travel intentions: an extended theory of planned behavior model based on the perception of COVID-19. *Tour. Rev.* 76 871–891. 10.1108/TR-09-2020-0458

[B59] Luo J. M. Lam C. F. (2020). Travel anxiety, risk attitude and travel intentions towards “Travel Bubble” destinations in Hong Kong: effect of the fear of COVID-19. *Int. J. Environ. Res. Public Health* 17:7859. 10.3390/ijerph17217859 33120949 PMC7672589

[B60] Luo J. M. Vu H. Q. Li G. Law R. (2020). Topic modelling for theme park online reviews: analysis of Disneyland. *J. Travel Tour. Mark.* 37 272–285. 10.1080/10548408.2020.1740138

[B61] Luo J. M. Vu H. Q. Li G. Law R. (2021). Understanding service attributes of robot hotels: a sentiment analysis of customer online reviews. *Int. J. Hosp. Manag.* 98:103032. 10.1016/j.ijhm.2021.103032

[B62] Ma J. Scott N. Gao J. Ding P. (2017). Delighted or satisfied? Positive emotional responses derived from theme park experiences. *J. Travel Tour. Mark.* 34 1–19. 10.1080/10548408.2015.1125824

[B63] Mamun M. A. (2021a). Exploring factors in fear of COVID-19 and its GIS-based nationwide distribution: the case of Bangladesh. *BJPsych Open* 7:5. 10.1192/bjo.2021.984 34407906 PMC8376996

[B64] Mamun M. A. (2021b). Suicide and suicidal behaviors in the context of COVID-19 pandemic in Bangladesh: a systematic review. *Psychol. Res. Behav. Manag.* 14:695. 10.2147/PRBM.S315760 34113185 PMC8185458

[B65] Matiza T. (2020). Post-COVID-19 crisis travel behaviour: towards mitigating the effects of perceived risk. *J. Tour. Futures* 10.1108/JTF-04-2020-0063 [Epub ahead of print].

[B66] Mbama C. I. Ezepue P. O. (2018). Digital banking, customer experience and bank financial performance. *Int. J. Bank Mark.* 36 230–255. 10.1108/IJBM-11-2016-0181

[B67] Mereu S. (2016). *Applying the Experience Economy Model to the Periscope Channel of a Football Club. Sports Business Research.* Available online at: https://sportsbusinessresearch.academy/2016/03/09/applying-the-experience-economy-model-to-the-periscope-channel-of-a-football-club/ (accessed February 14, 2022).

[B68] Miles A. Voorwinden S. Chapman S. Wardle J. (2008). Psychologic predictors of cancer information avoidance among older adults: the role of cancer fear and fatalism. *Cancer Epidemiol. Prev. Biomark.* 17 1872–1879. 10.1158/1055-9965 18708374

[B69] Mileva G. (2021). *Can Augmented Reality Help Theme Parks Recover After the Pandemic?.* Available online at: https://arpost.co/2021/01/05/augmented-reality-theme-parks-after-pandemic/ (accessed Aug 15, 2021).

[B70] Milman A. Tasci A. D. Wei W. (2020). Crowded and popular: the two sides of the coin affecting theme-park experience, satisfaction, and loyalty. *J. Destination Mark. Manag.* 18:100468. 10.1016/j.jdmm.2020.100468

[B71] Moreira P. (2008). Stealth risks and catastrophic risks: on risk perception and crisis recovery strategies. *J. Travel Tour. Mark.* 23 15–27. 10.1300/J073v23n02_02

[B72] Nawijn J. Fricke M. C. (2015). Visitor emotions and behavioral intentions: the case of concentration camp memorial Neuengamme. *Int. J. Tour. Res.* 17 221–228. 10.1002/jtr.1977

[B73] Nguyen C. Romaniuk J. (2014). Pass it on: a framework for classifying the content of. word of mouth. *Aust. Mark. J. (AMJ)* 22 117–124. 10.1016/j.ausmj.2013.12.014

[B74] Oh J. Ma H. (2018). Enhancing visitor experience of theme park attractions: focusing on animation and narrative. *J. Adv. Res. Dyn. Control Syst.* 10 178–185. 10.17758/EIRAI.DIRH1017012

[B75] Peng L. Zhang W. Wang X. Liang S. (2019). Moderating effects of time pressure on the relationship between perceived value and purchase intention in social E-commerce sales promotion: considering the impact of product involvement. *Inf. Manag.* 56 317–328. 10.1016/j.im.2018.11.007

[B76] Person B. Sy F. Holton K. Govert B. Liang A. (2004). Fear and stigma: the epidemic. within the SARS outbreak. *Emerg. Infect. Dis.* 10:358. 10.3201/eid1002.030750 15030713 PMC3322940

[B77] Pine B. J. Gilmore J. H. (1999). *The Experience Economy: Work is Theatre & Every Business a Stage.* Boston, MA: Harvard Business Press.

[B78] Prebensen N. K. Woo E. Chen J. S. Uysal M. (2013). Motivation and involvement as antecedents of the perceived value of the destination experience. *J. Travel Res.* 52 253–264. 10.1177/0047287512461181

[B79] Qi C. X. Gibson H. J. Zhang J. J. (2009). Perceptions of risk and travel intentions: the case of China and the Beijing Olympic Games. *J. Sport Tour.* 14 43–67. 10.1080/14775080902847439

[B80] Quadros S. Garg S. Ranjan R. Vijayasarathi G. Mamun M. A. (2021). Fear of COVID 19 infection across different cohorts: a scoping review. *Front. Psychiatry* 12:708430. 10.3389/fpsyt.2021.708430 34557117 PMC8453018

[B81] Rad R. E. Mohseni S. Takhti H. K. Azad M. H. Shahabi N. Aghamolaei T. (2021). Application of the protection motivation theory for predicting COVID-19 preventive behaviors in Hormozgan, Iran: a cross-sectional study. *BMC Public Health* 21:466. 10.1186/s12889-021-10500-w 33685426 PMC7938277

[B82] Rahimizhian S. Irani F. (2020). Contactless hospitality in a post-Covid-19 world. *Int. Hosp. Rev.* 35 293–304. 10.1108/IHR-08-2020-0041

[B83] Rather R. A. (2021a). Demystifying the effects of perceived risk and fear on customer engagement, co-creation and revisit intention during COVID-19: a protection motivation theory approach. *J. Destination Mark. Manag.* 20:100564. 10.1016/j.jdmm.2021.100564

[B84] Rather R. A. (2021b). Monitoring the impacts of tourism-based social media, risk perception and fear on tourist’s attitude and revisiting behaviour in the wake of COVID-19 pandemic. *Curr. Issues Tour.* 24 1–9. 10.1080/13683500.2021.1884666

[B85] Reisinger Y. Mavondo F. (2015). Travel anxiety and intentions to travel internationally: implications of travel risk perception. *J. Travel Res.* 43 212–225. 10.1177/0047287504272017

[B86] Rogers R. W. (1975). A protection motivation theory of fear appeals and attitude change1. *J. Psychol.* 91 93–114. 10.1080/00223980.1975.9915803 28136248

[B87] Sánchez-Cañizares S. M. Cabeza-Ramírez L. J. Muñoz-Fernández G. Fuentes-García F. J. (2021). Impact of the perceived risk from Covid-19 on intention to travel. *Current Issues in Tourism*, 24, 970–984. 10.1080/13683500.2020.1829571

[B88] Schroeder A. Pennington-Gray L. Kaplanidou K. Zhan F. (2013). Destination risk perceptions among US residents for London as the host city of the 2012 Summer Olympic Games. *Tour. Manag.* 38 107–119. 10.1016/j.tourman.2013.03.001

[B89] Shafiei A. Maleksaeidi H. (2020). Pro-environmental behavior of university students: application of protection motivation theory. *Glob. Ecol. Conserv.* 22:e00908. 10.1016/j.gecco.2020.e00908

[B90] Shaw G. Bailey A. Williams A. (2011). Aspects of service-dominant logic and its implications for tourism management: examples from the hotel industry. *Tour. Manag.* 32 207–214. 10.1016/j.tourman.2010.05.020

[B91] Shi J. Chen Z. Wang X. Teng F. Yang Y. Chen H. (2021). Dominate others, hurt self: social dominance orientation predicts depression during the COVID-19 pandemic. *Pers. Individ. Differ.* 175:110710. 10.1016/j.paid.2021.110710 34848904 PMC8613708

[B92] Sigala M. (2020). Tourism and COVID-19: impacts and implications for advancing and resetting industry and research. *J. Bus. Res.* 117 312–321. 10.1016/j.jbusres.2020.06.015 32546875 PMC7290228

[B93] Sniehotta F. F. Presseau J. Araújo-Soares V. (2014). Time to retire the theory of planned behaviour. *Health Psychol. Rev.* 8 1–7. 10.1080/17437199.2013.869710 25053004

[B94] Solmaz G. Akbaş M. Ý Turgut D. (2015). A mobility model of theme park visitors. *IEEE Trans. Mobile Comput.* 14 2406–2418. 10.1109/TMC.2015.2400454

[B95] Strong P. (1990). Epidemic psychology: a model. *Sociol. Health Illness* 12 249–259. 10.1111/1467-9566.ep11347150

[B96] Szymkowiak A. Gaczek P. Jeganathan K. Kulawik P. (2021). The impact of emotions on shopping behavior during epidemic. What a business can do to protect customers. *J. Consum. Behav.* 20 48–60. 10.1002/cb.1853 PMC7436385 38607848

[B97] Taber K. S. (2018). The use of Cronbach’s alpha when developing and reporting research instruments in science education. *Res. Sci. Educ.* 48 1273–1296. 10.1007/s11165-016-9602-2

[B98] TEA (2019). *Global Attractions Attendance Report.* Available online at: https://aecom.com/wp-content/uploads/2020/07/2019-Theme-Index-web-1.pdf (accessed February 14, 2022).

[B99] UNWTO (2020). *International Tourist Numbers Could Fall 60-80% in 2020, UNWTO Report.* Available online at: https://www.unwto.org/news/covid-19-international-tourist-numbers-could-fall-60-80-in-2020 (accessed April 25, 2021).

[B100] Wang J. Liu-Lastres B. Ritchie B. W. Mills D. J. (2019). Travellers’ self-protections against health risks: an application of the full Protection Motivation Theory. *Ann. Tour. Res.* 78:102743. 10.1016/j.annals.2019.102743

[B101] Wei W. Qi R. Zhang L. (2019). Effects of virtual reality on theme park visitors’ experience and behaviors: a presence perspective. *Tour. Manag.* 71 282–293. 10.1016/j.tourman.2018.10.024

[B102] Wen J. Kozak M. Yang S. Liu F. (2020). COVID-19: potential effects on Chinese citizens’ lifestyle and travel. *Tour. Rev.* 76 74–87. 10.1108/TR-03-2020-0110

[B103] WenJuanXing (2021). *About Us.* Available online at: http://www.wjx.cn/html/aboutus.aspx (accessed April 12, 2021).

[B104] Wu C. H. J. (2011). A re-examination of the antecedents and impact of customer participation in service. *Serv. Ind. J.* 31 863–876. 10.1080/02642060902960768

[B105] Xu J. Chan A. (2010). Service experience and package tours. *Asia Pac. J. Tour. Res.* 15 177–194. 10.1080/10941661003629987

[B106] Yang H. J. (2020). *With 80% Revenue Loss in the First Quarter and the Closure of Amusement Parks, Where is the “Joy” of Domestic Theme Parks?.* Available online at: https://travel.ifeng.com/c/7vi8sad0yNE (accessed April 25, 2021).

[B107] Ye H. B. Fong L. H. N. Luo J. M. (2021). Parasocial interaction on tourism companies’ social media sites: antecedents and consequences. *Curr. Issues Tour.* 24 1093–1108. 10.1080/13683500.2020.1764915

[B108] Zheng D. Luo Q. Ritchie B. W. (2021). Afraid to travel after COVID-19? Self-protection, coping and resilience against pandemic ‘travel fear’. *Tour. Manag.* 83:104261. 10.1016/j.tourman.2020.104261

